# Tau Positivity Did Not Correlate with PHQ9 Depression Scores in a Memory Clinic.

**DOI:** 10.1093/geroni/igaf122.2553

**Published:** 2025-12-31

**Authors:** Viviana Obando, Malini Nair, Rui Huang, Surya Sunil, Kavya Chemalapatti, Anil Nair

**Affiliations:** ADC, Braintree, Massachusetts, United States; ADC, Braintree, Massachusetts, United States; ADC, Braintree, Massachusetts, United States; ADC, Braintree, Massachusetts, United States; ADC, Braintree, Massachusetts, United States; ADC, Braintree, Massachusetts, United States

## Abstract

**Background:**

It is unknown if depression levels are correlated to positivity on tau blood or PET scan results in memory clinic patients.

**Method:**

We performed a retrospective analysis of memory clinic patients in the south shore of Boston from 2010 to 2025. We correlated depression screen data (PHQ9) to tau PET or Blood test positivity scores. Univariate analyses used Pearson correlation. A multivariate regression model analyzed PHQ9 to covariates of tau positivity, age, sex, education and race.

**Hypothesis:**

We hypothesized a positive correlation between depression levels scored by the PHQ9 and abnormal tau test results.

**Results:**

1043 patients attended the memory clinic between 2010-2025; 20 had PHQ9 and tau blood test results with analyzable data. Patients were 58.9% female, 85.1 % White. Mean age was 70±14.2 years, Tau score did not correlate significantly to PHQ9 Multivariate model confirmed the lack of association of depression scores to tau abnormality (100000= 0.08, p = 0.44).

**Conclusions:**

Depression scores using PHQ9 during screening were not affected by tau positivity as measured as a binary outcome during tau disclosure in a memory clinic.

